# Does team reflexivity impact teamwork and communication in interprofessional hospital-based healthcare teams? A systematic review and narrative synthesis

**DOI:** 10.1136/bmjqs-2019-009921

**Published:** 2020-01-07

**Authors:** Siobhan Kathleen McHugh, Rebecca Lawton, Jane Kathryn O'Hara, Laura Sheard

**Affiliations:** 1 School of Psychology, University of Leeds, Leeds, UK; 2 Yorkshire Quality and Safety Research Group, Bradford Institute for Health Research, Bradford, UK; 3 Leeds Institute of Medical Education, University of Leeds, Leeds, UK

**Keywords:** Teamwork, communication, reflexivity, healthcare quality improvement, healthcare staff

## Abstract

**Background:**

Teamwork and communication are recognised as key contributors to safe and high-quality patient care. Interventions targeting process and relational aspects of care may therefore provide patient safety solutions that reflect the complex nature of healthcare. Team reflexivity is one such approach with the potential to support improvements in communication and teamwork, where reflexivity is defined as the ability to pay critical attention to individual and team practices with reference to social and contextual information.

**Objective:**

To systematically review articles that describe the use of team reflexivity in interprofessional hospital-based healthcare teams.

**Methods:**

Following the Preferred Reporting Items for Systematic Reviews and Meta-Analyses guidelines, six electronic databases were searched to identify literature investigating the use of team reflexivity in interprofessional hospital-based healthcare teams.

The review includes articles investigating the use of team reflexivity to improve teamwork and communication in any naturally occurring hospital-based healthcare teams. Articles’ eligibility was validated by two second reviewers (5%).

**Results:**

Fifteen empirical articles were included in the review. Simulation training and video-reflexive ethnography (VRE) were the most commonly used forms of team reflexivity. Included articles focused on the use of reflexive interventions to improve teamwork and communication within interprofessional healthcare teams. Communication during interprofessional teamworking was the most prominent focus of improvement methods. The nature of this review only allows assessment of team reflexivity as an activity embedded within specific methods. Poorly defined methodological information relating to reflexivity in the reviewed studies made it difficult to draw conclusive evidence about the impact of reflexivity alone.

**Conclusion:**

The reviewed literature suggests that VRE is well placed to provide more locally appropriate solutions to contributory patient safety factors, ranging from individual and social learning to improvements in practices and systems.

**Trial registration number:**

CRD42017055602.

## Background

Traditionally, measurement of and intervention for patient safety have focused on learning from specific harm events. The effectiveness of this approach is limited, relying, as it does, on retrospective reports and producing recommendations for practice based on unrealistic views of in-situ clinical work.^1^ Further challenges include engagement of front-line staff and insufficient attention given to complexity within healthcare systems.^1–3^

By definition, complexity concerns *‘the interrelatedness of the components within a system’*,^4 5^ or how the components within a system influence each other. As the number of components increases (eg, increasing patient numbers, interprofessional working and levels of care), the complexity of the system will increase. There is growing recognition that quality improvement approaches must account for this increasing complexity^6^ and the emergence of more transient, interprofessional teams.^7^ However, training continues to occur largely within discipline-specific groups, often leading to the development of hierarchical systems or silos.^8^ Consequently, failures in teamwork and communication have been identified as substantial contributors to medical error and compromised patient safety.^8–11^ Thus, interventions targeting such process and relational aspects of care may provide patient safety solutions that reflect the complex nature of healthcare. Team reflexivity is one such approach with potential to support improvements in communication and teamwork.

### Reflexivity and healthcare teams

Reflexivity is seen as a way to frame individual actions and behaviours with reference to the effect of the actions and behaviours of others, and the context in which these actions occur.^12 13^ Reflexive methods assume that awareness of self within teams, systems or organisations is key to developing distributed intelligence and the potential for locally appropriate solutions.^7 14^ This is differentiated from reflection, where individual actions are considered more distantly, in the absence of context.^13^

Reflexivity as a collective practice in healthcare^7 15^ is less well established and researched than individual reflection.^16–18^ However, it is argued to be appropriate for teams of healthcare practitioners to consider routine practices based on contextual and situational factors.^7 19^ Moreover, research focusing on improvements in non-technical skills (NTS) within healthcare teams has seen the concept of reflexive practice becoming embedded within peer review^20 21^ and simulation training.^22 23^ Team reflexivity in this context is most commonly delivered via a debriefing session, during which group discussion of both technical and non-technical skills is facilitated or prompted within group. As with simulation training and peer review more generally, team reflexivity in this form is often problem-centred or task-specific.^24^

A more novel approach to team reflexivity is video-reflexive ethnography (VRE).^14 25^ This involves filming specific interactions or practices in situ and replaying appropriate clips to staff teams. Presentation of in-situ footage is suggested to make explicit to practitioners what they do to accomplish safe patient care within the inherent complexities of healthcare.^14 26 27^ Making routine practices explicit allows teams to shift away from specificity and talk at increasingly higher levels of generality to identify commonly occurring features in their working practices, and to develop a common ground on how to organise and manage these practices collectively.^13 14 28–30^

This review therefore aims to collate the literature exploring the use of reflexivity in interprofessional healthcare teams, specifically attempting to understand the use of reflexivity in interprofessional teams working in the provision of hospital-based healthcare and how these tools might impact patient safety.

The review will focus on the following questions:

- How has reflexivity been used with interprofessional healthcare teams?
- How do staff respond to different methods of supporting team reflexivity?
- Does team reflexivity work to effect change in teamwork and communication?

## Methods

### Search strategy

This review was guided by the Preferred Reporting Items for Systematic Reviews and Meta-Analyses statement (see online supplementary appendix A),^31^ and the protocol was published on PROSPERO (International Prospective Register of Systematic Reviews). Search terms including “reflexiv*”, “video ADJ1 feedback”, “simulat* training” or “peer assess*” identified articles relating to reflexive methods. These were combined with terms to identify hospital-based multidisciplinary teams. The search strategy was applied to PsycINFO, Ovid MEDLINE, PubMed, Web of Science, Cochrane Library and Cumulative Index to Nursing and Allied Health Literature in January 2017 and updated in November 2017. Only studies published in the English language were included due to limited translation resources. Searches were limited to retrieve articles published after 1990 where the databases allow. The use of reflexivity in healthcare is a focal area of research with a small number of research groups. To identify further studies in publication that might meet the inclusion criteria, the lead authors in these groups were contacted. The academic search strategies and full results of all searches are detailed in online supplementary appendix B.

10.1136/bmjqs-2019-009921.supp1 Supplementary data

10.1136/bmjqs-2019-009921.supp2 Supplementary data

### Eligibility criteria and study selection

The inclusion criteria are outlined in table 1. A single reviewer (SKM) screened the titles and abstracts and conducted a full-text review. A subsample of articles were independently reviewed by CW (10%; n=256). Inter-rater reliability was assessed using Cohen’s kappa, and strong agreement on inclusion and exclusion of papers for full-text review was found (k=0.92). Regular meetings with the second reviewer allowed discussion of article eligibility. Four hundred and one articles were selected for full-text review, of which 5% were second-reviewed independently by RL and JKOH (n=20). Inter-rater reliability was assessed using Cohen’s kappa.^32^ Strong agreement (k=0.84) was found for the full-text review. Discrepancies were resolved through discussion between the reviewers. Reasons for exclusion were recorded.

**Table 1:** Eligibility criteria for inclusion of academic articles in the review

PICOS	Eligibility criteria
Population	Any naturally occurring hospital-based healthcare teams, where a team is defined as ‘*two or more healthcare professionals linked in a common purpose*’. Teams must be interdisciplinary. Any study including healthcare teams working outside of a hospital were excluded.
Intervention	Any studies using reflexivity, including (but not limited to) video reflexivity and video-reflexive ethnography. Reflexivity is defined as ‘*a tool that allows broader attention to routine working practices, providing renewed awareness and facilitated or prompted discussion of taken-as-given processes and interactions*’. Reflexivity is not a linear or rigid framework or method, but a more creative and flexible approach to understanding and reshaping practice through space for collective discussion.
Comparison	Not relevant.
Outcomes	Any measure or discussion of change in knowledge, attitudes, feelings/emotions and behaviours. Any measure or discussion of impact on teamwork, interprofessional communication and collective values. Any measure of improvement in efficiency of working practice, quality of care or patient safety. Any measure of outcomes associated with the success of healthcare delivery within a hospital. Any evaluation or discussion of the quality of reflexivity as an intervention.
Study design	Any peer-reviewed, academic articles using any empirical study design were included. Qualitative, quantitative and mixed-methods studies were included.

### Assessment of study quality

Study quality was assessed using the Quality Assessment Tool for Studies with Diverse Designs (QATSDD).^33^ The QATSDD is a validated quality assessment tool for use with methodologically heterogeneous studies, using 16 items on a 4-point Likert scale. Included studies were scored and study quality expressed as a percentage. SKM conducted quality assessments for all studies. Quality assessment was independently reviewed by RL (20%, n=3) and agreement on scores was found to be 100%. Any queries about quality assessment scores, where the primary reviewer (SKM) felt the score was on a boundary, were resolved by discussion with RL, JKOH and LS.

### Data extraction and synthesis

All data were extracted by a single researcher (SKM) using predefined data extraction points (online supplementary appendix C). Following the UK Economic and Social Research Council guidance,^34^ narrative synthesis was used due to the heterogeneous nature of the studies. This allowed for comments on study design, context and quality according to standard format, but also allowed similarities and differences to be explored between heterogeneous study designs.^35^ Preliminary themes were developed through the data extraction process using categories, clusters and brief textual descriptions addressing the specific research questions identified in this review. Results are presented under grouped headings related to the specific research questions addressed in this review.

10.1136/bmjqs-2019-009921.supp3 Supplementary data

## Results

**Figure 1:**
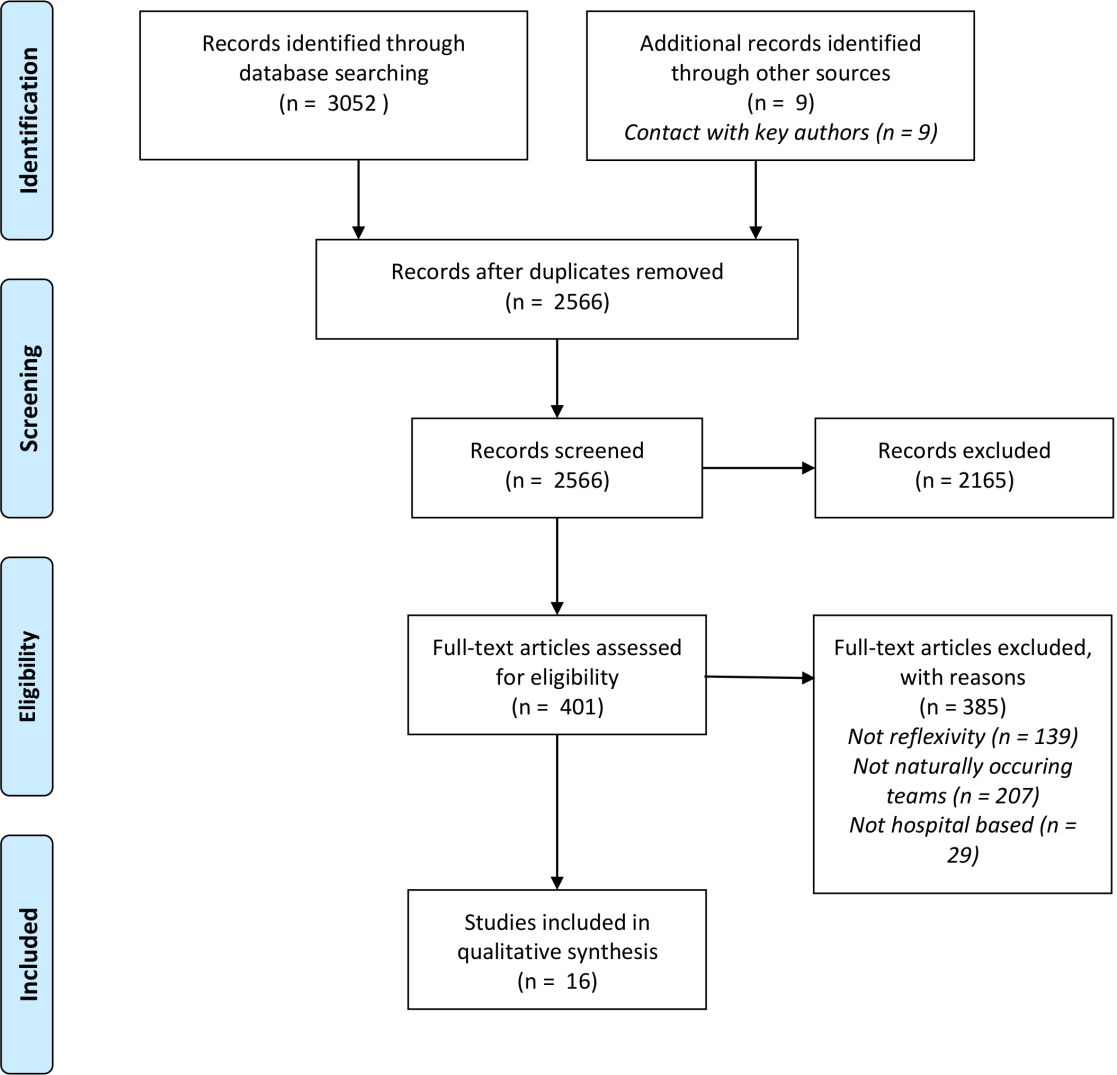
PRISMA flow diagram.^31^ PRISMA, Preferred Reporting Items for Systematic Reviews and Meta-Analyses.

The search strategy yielded 2566 articles excluding duplicates. In total, 15 articles met the inclusion criteria and were included in the review (figure 1). Articles were primarily excluded for not explicitly working with naturally occurring interprofessional teams or where feedback methods did not align with the definition of reflexivity outlined (table 1). The key characteristics of the included articles are outlined in online supplementary appendix D. Simulation training and VRE were the most commonly used forms of team reflexivity. It was also applied within reciprocal peer review. All included articles were set in high-risk hospital environments and set out to engender optimisation of daily practice^25 36–38^; evaluate specific reflexive methods as quality improvement strategies^39–45^; and/or develop NTS to improve safe and effective working practice^46–49^ (table 2). All included articles were published between 2006 and 2017.

10.1136/bmjqs-2019-009921.supp4 Supplementary data

**Table 2:** Article settings and team types

Author	Setting	Team type	Team size/composition
Allan *et al* 46	24-bed dedicated paediatric cardiac intensive care unit (USA).	Paediatric cardiac intensive care teams.	Nurses (n=127). Cardiology, cardiac surgery and cardiac critical care fellows (n=44). Paediatric cardiac intensive care unit attending physicians (n=6). Respiratory therapists (n=2). Nurse practitioners (n=3).
Aveling *et al* 39	Lung cancer teams in 30 National Health Service hospitals (UK).	Lung cancer teams.	Minimum requirement of: A clinical lead (physician). A clinical nurse specialist. A multidisciplinary team coordinator.
Carroll *et al* 25	Intensive care unit in a tertiary referral and teaching hospital (Australia).	Intensive care unit teams.	Included clinical specialists, specialist intensivists, nurses and allied health professionals.
Falcone *et al* 40	Paediatric trauma unit in level 1 paediatric trauma centre (USA).	Paediatric trauma teams.	An average team of around 6 members from: Paediatric surgeons (n=11). Emergency medics (n=7). Surgical residents (n=72). Nurses (n=60). Critical care fellows (n=4). Paramedics (n=2). Respiratory therapists (n=4).
Fransen *et al* 47	Obstetric unit (The Netherlands).	Multiprofessional obstetric teams.	Included gynaecologists, obstetricians, secondary care midwives and/or resident nurses.
Hor *et al* 36	Two general intensive care units in a major metropolitan teaching hospital (Australia).	Intensive care unit staff teams.	Included senior and junior doctors, senior and junior nurses, medical and nurse managers, ward clerks, receptionists, and allied health professionals.
Iedema *et al* 37	Emergency departments of two large teaching hospitals (one metropolitan, one regional; Australia).	Emergency department staff.	Paramedics, emergency department medics and nursing clinicians.
Iedema and Carroll^41^	Acute outpatient spinal clinic in a local metropolitan teaching hospital (Australia).	Multidisciplinary care team.	Doctors, nurses, occupational therapists, physiotherapists, dietitians, social workers and peer support workers.
Iedema *et al* 38	Intensive care unit and mixed surgical wards in two metropolitan teaching hospitals (Australia).	Intensive care unit and surgical ward staff.	107 nurses, 44 doctors, 9 allied health professionals and 17 administration and cleaning staff.
Iedema *et al* 42	Acute outpatient spinal pressure area clinic in a local metropolitan teaching hospital (Australia).	Outpatient unit teams.	Medical, nursing and allied health staff.
Iedema *et al* 43	Intensive care unit (Australia).	Intensive care unit staff.	Multidisciplinary teams of healthcare practitioners. Make-up of the teams unspecified.
Lehner *et al* 44	Paediatric trauma unit (Germany).	Paediatric trauma unit.	14 physicians including paediatric surgeons, intensivists, emergency medics and anaesthetists. 4 paediatric nurses.
Patterson *et al* 45	Paediatric emergency department (USA).	Paediatric emergency department.	Physicians: 51%. Nurses: 32%. Paramedics: 4%. Respiratory therapists: 3%. Patient care assistant: 4%. Other: 7%.
Patterson *et al* 48	Level 1 paediatric trauma centre (USA).	All healthcare providers in emergency department.	Faculty and staff physicians, nurses, respiratory therapists, paramedics, patient care assistants, and medical residents.
Ross *et al* 49	Tertiary hospital trust providing a range of specialist older persons services (UK).	Staff involved in the provision of elderly care.	Healthcare assistants, nurses, physiotherapists and medical staff.

### Quality assessment

The quality of studies was variable, with total scores ranging from 40% to 83% (mean=60%; online supplementary appendix E). Few studies justified the sample size, data collection methods or methods of data analysis. There was limited discussion of relevant theories to guide the methods used. Detailed recruitment information was not well reported; for example, most studies using videos did not provide appropriate detail of the process of consent or what would happen if members of a team did not provide consent.^25 36 37 40–44 46–49^

10.1136/bmjqs-2019-009921.supp5 Supplementary data

Limited detail regarding specific elements of team reflexivity made it difficult to determine how reflexive feedback was delivered. This was particularly true of the facilitation of feedback and how the feedback session itself was structured.^37 39–49^ There was limited detail regarding the methods used to analyse the effect of team reflexivity specifically with respect to NTS in most articles,^25 37–39 41–43 45^ making it difficult to learn about what works and what does not.

### Reflexivity in interprofessional healthcare teams

Three methods currently promoting reflexive practice in interprofessional healthcare teams were identified from the reviewed articles: team debrief postsimulation,^40 44–49^ reciprocal peer review^39^ and VRE.^25 36–38 41–43^ While the aims of these interventions are consistent, differences were apparent with respect to data collection and outcome measures. Simulation studies generally used quantitative outcome measures, and studies of in-situ methods generally used qualitative data. Table 3 summarises the key reflexive features of all studies.

**Table 3:** Key features of the reflexive elements of each study

Author	Aim of reflexivity	Facilitation	Duration of reflexive feedback
Allan *et al* 46	To uncover system faults or cognitive processes leading to suboptimal teamwork.	Trained physician and nurse facilitators. *Video footage used.*	No information provided.
Aveling *et al* 39	To allow a safe space to share challenges and working practices, and generate locally appropriate solutions.	External non-clinical facilitator.	No information provided.
Carroll *et al* 25	To engage healthcare professionals in problem-solving their own communication difficulties.	Researcher facilitation. *Video footage used.*	90 min.
Falcone *et al* 40	To emphasise team performance and communication, and reinforce appropriate care principles.	No information provided. *Video footage used.*	30 min.
Fransen *et al* 47	To allow deeper analysis of performance by group discussion.	Two facilitators. *Video footage used.*	30 min.
Hor *et al* 36	To provide space for discussion of how clinical spaces impacted on communication practices.	Researcher-facilitated. *Video footage used.*	No information provided.
Iedema *et al* 37	To form and articulate views about what is essential information that must be communicated and what are the critical processes involved in the handover.	No information provided about facilitation. *Video footage used.*	60–90 min.
Iedema and Carroll^41^	To capture staff insights and ideas to strengthen the organisational and communicative dimensions of healthcare provision.	No information provided about facilitation. *Video footage used.*	No information provided.
Iedema *et al* 38	To allow practitioners the space to raise questions about taken-for-granted infection control behaviours and scrutinise their own practice.	No information provided about facilitation. *Video footage used.*	No information provided.
Iedema *et al* 42	To allow staff to identify and address previously unrecognised environmental risk factors.	No information provided about facilitation. *Video footage used.*	No information provided.
Iedema *et al* 43	To discuss and address the strengths and weaknesses of handover practice.	Researcher facilitation. *Video footage used.*	No information provided.
Lehner *et al* 44	To evaluate and improve communication practices during paediatric trauma incidents.	Two-person multidisciplinary and multiprofessional instructor team. *Video footage used.*	45 min.
Patterson *et al* 45	To identify latent safety threats and subsequent multidisciplinary problem-solving.	Group assessment of performance.	10 min.
Patterson *et al* 48	To improve situational awareness and sharing of shared mental models.	Researcher facilitation. *Video footage used.*	No information provided.
Ross *et al* 49	To focus on non-technical skills including communication and improving a shared mental model.	Clinicians and trained professionals from a dedicated simulation centre.	45 min.

In seven studies, team reflexivity was embedded in simulation training programmes as collective debriefing. Teams were asked to participate in simulated practices replicating regularly occurring real-world emergencies^40 44–48^ or routine care practices.^49^ Simulating scenarios allowed staff to develop and refine skills and practices without the risk of causing harm to patients, to focus on their role within the team, and on how the team worked together to achieve specific clinical outcomes.

In eight studies, reflexive discussion was prompted following in-situ observation. Seven of these studies employed VRE to effect reflexive discussion.^25 36–38 41–43^ In the remaining study, staff teams heard peer observations of daily practice prompting reflexive discussion of issues and solutions.^39^ The purpose of collective reflexive discussions in both cases was to allow staff to confront the complexities of in-situ practice and the space to discuss locally appropriate solutions.

Reviewed studies generally lacked information about the role of the facilitator in prompting reflexive discussion. Studies refer to the facilitator as being a researcher or an independent healthcare professional, but there is no exploration of their role within the reflexive feedback session.

Studies included in this review generally lacked theoretical underpinning, making it difficult to gain insight into the active components of reflexivity. Although most studies used videos to prompt reflexive discussion, only four studies engaged briefly in the theory of this, suggesting that viewing routine practice can promote individual and collective learning.^25 36 42 43^ Only Iedema *et al* 38 made specific reference to adult learning theory linked to learning from reflexive feedback.

### Staff response to team reflexivity

Reflexive feedback appears to be accepted as successful in allowing staff to explore the intrinsic complexities of their daily work and develop technical and non-technical skills.^25 36–39^ However, only one study directly conveyed staff evaluation of the feedback sessions,^44^ reporting 100% of staff participants found the feedback sessions useful to inform their clinical practice.

The use of video in prompting reflexive discussion is less well defined in terms of staff acceptability and research feasibility. Iedema *et al* 42 reported staff discomfort with the potentially intrusive nature of the camera, and two studies identified the use of video footage as a potential barrier to staff engagement due to assumptions of professional judgement.^41 42^ Conversely, three studies^25 41 44^ reported that staff identified video footage as fundamental in allowing them to view daily practice and identify areas to improve.

### Team reflexivity to effect change in teamwork and interprofessional communication

Communication during interprofessional teamworking was the most prominent focus of improvement methods,^25 37 39 41–44 46 48 49^ although studies also focused on environmental or process improvements,^36 38^ and improvements in specific patient safety measures resulting from learning about communication and collective working.^45 47^ The data collection methods of all studies are outlined briefly in table 4.

**Table 4:** Key data collection methods of the reviewed articles

Author	Quantitative measures	Qualitative data
Allan *et al* 46	Precourse and postcourse programme evaluation questionnaires.	
Aveling *et al* 39		Non-participant observation, interviews and documentary analysis.
Carroll *et al* 25		Ethnographic observations, video footage of reflexive feedback sessions.
Falcone *et al* 40	Multidisciplinary team simulation evaluation tool.	
Fransen *et al* 47	Composite outcome of low Apgar score, severe postpartum haemorrhage, trauma due to shoulder dystocia, eclampsia and hypoxic-ischaemic encephalopathy.	
Hor *et al* 36		Semistructured interviews, ethnographic observations and reflexive focus groups.
Iedema *et al* 37	Preimplementation and postimplementation surveys to measure nurse perceptions of new handover protocol. Analysis of video footage scored on specific categories proposed by emergency department clinicians.	Focus groups, ethnographic observation and reflexive focus groups.
Iedema and Carroll^41^		Interviews, documentary analysis, ethnographic observations and reflexive feedback sessions.
Iedema *et al* 38		Interviews, ethnographic observations and reflexive feedback sessions.
Iedema *et al* 42	Analysis of unit spending costs per patient admission.	Interviews, ethnographic observations and reflexive feedback sessions.
Iedema *et al* 43		Focus groups, ethnographic observations and reflexive feedback meetings.
Lehner *et al* 44	Precourse and postcourse evaluation surveys.	
Patterson *et al* 45	Number and type of latent safety threats identified during simulations. Blinded video review of teamwork behaviours using a modified Anaesthetists Non-Technical Skills scale. Electronic survey to measure participant assessment of the course.	
Patterson *et al* 48	Number of days without a patient safety event in the emergency department. Knowledge tests at baseline, postintervention and re-evaluation, Safety Attitudes Questionnaire scores.	
Ross *et al* 49	Premodule and postmodule questionnaire scores to assess participant self-confidence.	Simulation observations and follow-up staff interviews post-training.

The following sections identify the main areas of improvement reported in the reviewed studies and how they were measured.

### Communication and teamwork

Eleven reviewed articles identified communication within interprofessional teams as a specific area of focus.^25 37 39 41–46 48 49^ Iedema *et al* 37 reported staff perception of improvements in information transfer during paramedic to emergency department handover following the codesign of a new protocol. The amount of information transferred reportedly increased (from 50% to 60%), but there was notably a sharp reduction in repetition of information (from 67% to 33%). Outcomes were obtained primarily through formal video analysis of preintervention and postintervention handovers and a staff survey gauging perception of the new handover protocol. Carroll *et al* 25 observed more concise and structured dissemination of information by nurses and doctors during intensive care unit handover following identification of issues in information transfer prompted by VRE. Iedema *et al* 43 reported perceived improvement nurse engagement during interprofessional clinical discussions, although staff still identified the need for refinement of new bedside handover interactions. Improvements in both studies^25 43^ were reported following observations pre-VRE and post-VRE and unstructured discussions with staff. Patterson *et al* 48 reported modification of communication behaviours following review of video footage by independent reviewers, using a modified version of the Behavioral Markers for Neonatal Resuscitation Scale to assess teamwork and communication preintervention and postintervention. This was the only study to link modification of team behaviours directly to patient safety outcomes, reporting a reduction in patient safety events from two or three per year to a period of over 1000 days without a patient safety event following the introduction of the training. Perceived improvement in reliable and effective communication was also reported on anonymised self-evaluation questionnaires in paediatric trauma teams following simulation-based team training, although comparison of preintervention and postintervention scores was not found to be significant.^44^ Although Patterson *et al* 45 reported no explicit improvement in teamwork behaviours over time, more general changes to structure and culture were observed, with the shared mental model identified as being so crucial to teamwork behaviours that staff asked for this to be added to the resuscitation flow sheet to be communicated to the team within the first 5 min of caring for a critically ill patient.

The remaining articles identified improved discourse within teams relating to changes in process or structure. Two studies identified development of discourse about the complexities of existing processes and collective rethinking of routine communication practices.^41 42^ Although there were clear narratives about the benefits of VRE in allowing teams to articulate the complexities and dynamism of healthcare pathways in these two articles, there was no formal measure of communication or of any specific process improvement in either study. Aveling *et al* 39 also provided clear discussion of the benefits of reciprocal peer review to allow staff to discuss issues and develop solutions, but this was not formally linked to improvements in communication. All three studies relied solely on unstructured discussion with staff and ethnographic observations, although their primary aim was to develop the use of more novel methods in patient safety research as opposed to specific practice improvement.

Ross *et al* 49 highlighted perceived improvements in interprofessional communication during clinical tasks, reporting strengthened teamwork and better communication between staff. Allan *et al* 46 also reported significantly increased likelihood of speaking up in the case of perceived inappropriate management of resuscitation events following subgroup analysis of self-report surveys preintervention and postintervention (p<0.001).

### Process improvements

Iedema *et al* 38 reported discussion and formulation of safer ways of dealing with infection risks and infection control practices. Site-specific improvements included appointing a single staff member to prevent any contact between gowned and gloved clinicians and infected patients, other clinicians and ward equipment. Findings were reported based on detailed ethnographic observations, related field notes and data gathered from staff interviews. Hor *et al* 36 also reported implementation of improved and flexible working spaces in intensive care units following video-facilitated reflexive feedback groups. Improvements focused particularly on the prevention of interruptions, such as doctors finding a quieter and more isolated space to prevent interruptions during weekly X-ray rounds. Both studies focused on structural changes, highlighting the importance of safe working spaces in enabling safer patient care and more effective teamwork.

### Safety outcomes

Two studies reported reflexive practice as a catalyst for improvement of safety. Fransen *et al* 47 reported reduction in trauma due to shoulder dystocia (from 0.25% to 0.16%) and increased levels of appropriate treatment for massive postpartum haemorrhage (0.28% vs 0.13%) following simulation designed to improve interprofessional teamwork during routine obstetric trauma. Patterson *et al* 45 reported improved identification of latent safety threats (LSTs) during in-situ emergency trauma simulations (1 LST for every 1.2 simulations) when compared with lab-based simulation training (1 LST for every 7 simulations).

Improvement in collective clinical confidence was reported following team debrief focused on the effectiveness of teamwork as well as technical skills.^45^

## Discussion

The current systematic review explores how reflexivity has been used to target factors contributing to patient safety within interprofessional healthcare teams. Although we focus on team reflexivity as a tool for the improvement of teamwork and communication in interprofessional healthcare teams, it is evident that the impact of team reflexivity also extends to improvements in specific clinical routine practices and clinical processes and to specific patient safety outcomes.

### The use of team reflexivity in healthcare

The use of reflexivity within interprofessional teams in healthcare research is becoming more widespread, reflecting the increasing complexities of safe and high-quality care. Three methods of prompting team reflexivity were identified in this review—team simulation training, reciprocal peer review and VRE—although papers differed in the way reflexivity was defined. Team reflexivity embedded within a wider simulation training or peer-review programme was often referred to as *‘team debrief’* or *‘team feedback’*. Nevertheless, collective discussion sessions across all methods aligned with accepted definitions of reflexivity in healthcare research.^14 30 50^

Going forward, a more detailed understanding of how team reflexivity works will be important in relation to learning and improvement in healthcare. Continued professional education methods, including simulation training and peer review, are grounded in an extensive body of theoretical literature, exploring situated learning through interaction as social psychological determinant of collective learning.^51^ Although more recent literature draws on complexity theory and the concept of psychological safety underpinning VRE as a collective learning tool and improvement method,^52^ there must be continued focus on exploring the factors that impact collective learning from the viewing of in-situ practice.^53^

Interpreting VRE methods through the lens of complexity theory^4 54–57^ accepts the importance of personal interactions and social influences on learning within dynamic and flexible environments such as healthcare.^51 57 58^ Drawing on social cognitive theories, transformative learning occurs when learners can question existing knowledge of processes, systems and interactions, and the underlying beliefs and assumptions.^58^ Iedema^50^ proposes that it is the de-familiarisation effect of video footage that allows participants to ask questions of themselves and others in context that defines VRE, presenting clear links to transformative learning, specifically transformation of individual and group perspectives.

Understanding how team reflexivity works must also extend to the role of the facilitator in prompting collective learning. There is limited reference to the role of the facilitator within the reviewed literature, despite good evidence from other research that the role of the facilitator is linked to successful reflective practice and collective learning.^52 59 60^ Further, there is emerging evidence to suggest the importance of the facilitator in the success of collaborative or sociocultural improvement methods in the healthcare literature.^61 62^

Finally, there is no exploration in the reviewed literature of whether the impact of reflexivity differs between teams and the factors that might affect the process of collective reflexive discussion. Exploration of the relevant literature suggests that high levels of psychological safety are significantly associated with more creative team performance and help teams to engage in learning behaviours due to reduced anxiety and a greater willingness to honestly share knowledge that requires risk.^63 64^ Future work should explore the role, composition and culture of the team and how these factors could potentially contribute to any outcomes of collective reflexive discussion.

### Staff perceptions

The majority of studies in this review explored the impact of team reflexivity or evaluated the methods used. Few studies investigated the acceptability of team reflexivity among staff, and it is unclear from the reviewed literature whether there are any issues of feasibility with reflexive methods in hospital-based healthcare teams. It is also uncertain whether the limited number of studies in this field reflects the infancy of the concept or the difficulty of using this approach within acute healthcare environments.

### Outcomes of team reflexivity

Two divergent observations emerged in this review regarding outcomes of team reflexivity. Studies of simulation training, by design, excised elements of complexity to focus specifically on the improvement of specific clinical procedures and the NTS aligned with such procedures. Conversely, improvement methods capturing in-situ practices and interactions, such as VRE, operate within the inherent complexities of healthcare provision. As such, articles focused on simulation training methods were of higher quality, predominantly due to the level of methodological and analytical detail provided, resulting in well-defined measures of change or improvement. However, evaluation of the reflexive feedback component was not isolated from other elements of the training; thus, any reported improvement in NTS could not be attributed solely to reflexive feedback.

Establishing the effectiveness of more adaptive, sociocultural interventions like VRE is more complex, with conventional approaches to evaluation less likely to be appropriate. Reviewed studies generally relied on ethnographic observations and unstructured discussions with staff to identify change or improvement; evaluation methods better placed to capture and account for complexity. Current literature suggests encouragement of methods prompting the development of flexible and locally appropriate goals and solutions should be embraced.^65^ The varied outcomes identified across reviewed studies suggest wide-ranging impact is possible where interventions engage with the complexities of acute healthcare practice.

### Review limitations

Poorly defined methodological information relating specifically to the reflexive elements of reviewed studies made it difficult to draw conclusive evidence about the impact of reflexivity alone. It is possible that simulation training, peer review and VRE would trigger individuals to reflect privately on the social and contextual underpinning of collective processes even in the absence of structured team reflexivity. The nature of this review only allows assessment of team reflexivity as an activity embedded within these methods.

Despite the application of an inclusive search strategy, relevant articles may not have been identified. Articles may not have referred to team reflexivity, specifically where collective feedback was embedded within wider improvement methods.

### Implications and recommendations

Healthcare professionals are often best placed to suggest change or improvement to working practices. Intuitively, it makes sense for staff to be empowered to identify and make these changes. The reviewed literature suggests that simulation training imposes simplicity on complex practices,^50^ thereby providing less opportunity for staff input in change or improvement within their discipline due to the focus of discussion being restricted to specific scenarios, although embedding reflexive feedback allows integration of NTS development into more established clinical training methods. Reciprocal peer review provided more opportunity for staff to discuss change or improvement at a process or systems level to some extent; however, quality improvement plans were based on team meetings on observations to provide local context supplemented by patient experience and audit data. Staff were thus provided the space and opportunity to discuss issues and potential solutions, although feedback on NTS within teams was dependent on individual and peer opinions or memories, and implementation of change was highlighted as requiring significant support. VRE is unique in its use of video footage to explore ‘real-time’ unfolding of specific healthcare practice, making explicit the complexity and dynamism of healthcare provision.^14^ Outcomes are less dependent on individual opinions or memories, but on how healthcare professionals individually and collectively respond to the footage. The reviewed literature suggests that all methods of team reflexivity have some impact on the improvement of contributory patient safety factors such as teamwork and communication. Furthermore, emerging literature suggests that VRE is best placed to empower participants to implement change and optimise processes or working environments, as well as allowing teams to learn together about the complexities of their daily interactions and routine practices.^66^

Importantly, the reviewed literature has highlighted particular areas for improvement relating to the study of team reflexivity in healthcare and the reporting of findings. It is important that future studies aim to justify their use of team reflexivity with reference to the theoretical foundations of the specific tool or intervention to be used, allowing authors to account for and provide detail of methodological and analytical choices. Studies must consider the acceptability of such methods in varied healthcare environments and must account for any issues of feasibility where they arise. Authors must focus on providing adequate description of the reflexive element of any study, including the context of the reflexive session and the level of facilitation. Analytical methods used must not only be detailed within the study method, but authors must also provide clear justification for their choices. Future studies should focus on analysis of the specific impact of reflexivity on NTS so that stronger conclusions can be made about the link between teams having the time and space to practise reflexivity, and subsequent improvements in these contributory patient safety factors.

## Conclusions

Reflexivity has been identified as a practice that encourages healthcare professionals to focus on improvements in the process and relational aspects of care, with high-fidelity team simulation training, team peer-review methods and VRE gradually becoming documented as improvement methods. The reviewed literature, combined with supporting literature in non-hospital-based care,^67 68^ suggests that VRE is well placed to provide more locally appropriate solutions to contributory patient safety factors, ranging from individual and social learning, to improvements in practices and systems. Thus, a continued focus on high-quality research and reporting is required to explore how this method can be integrated into acute, high-risk organisations, and particularly how reflexive discussion can be prompted within often transient interprofessional teams to promote interprofessional learning and optimisation of routine practices.
