# Supplementary material for: Does team reflexivity impact teamwork and communication in interprofessional hospital-based healthcare teams? A systematic review and narrative synthesis
Source: BMJ Qual Saf. 2020 Jan 7;29(8):672–83. doi: 10.1136/bmjqs-2019-009921 (PMC7398296; doi:10.1136/bmjqs-2019-009921)
Supplement: Supplementary data [file bmjqs-2019-009921supp002.pdf]

Database: Ovid MEDLINE(R) <1996 to 2016>

Search strategy:

1	reflexiv* (3174)
2	video ADJ1 feedback (247)
3	ethnograph* (6438)
4	critical reflect* (705)
5	peer assessment* (MeSH ‘peer review, healthcare’) (1210)
6	simulation* (MeSH ‘simulation training/mt, og) (448)
7	1 OR 2 OR 3 OR 4 OR 5 OR 6 or 7 (11853)
8	hospital ADJ1 team* (213)
9	multi-disciplinary team* (1734)
10	ward* (MeSH ‘health personnel/ed, og, px, st, td’) (11497)
11	hospital ADJ1 unit* (7209)
12	hospital ADJ1 department* (16213)
13	hospital* (MeSH ‘Hospital-patient relations/ or Medical staff, Hospital/ or Hospital-physician relations/ or Nursing staff, Hospital/ or Personnel, Hospital/ or Hospital Communication Systems (43215)
14	9 OR 10 OR 11 OR 12 OR 13 OR 14 (77664)
15	8 AND 15 (585)

Database: PsychINFO <2002 to 2016>

Search strategy:

1	reflexiv* (6327)
2	video ADJ1 feedback (356)
3	ethnograph* (19449)
4	critical reflect* (1699)
5	peer assessment* (MeSH ‘exp Medical Education/ and exp Peer Evaluation/) (47)
6	simulation* (186)
7	1 OR 2 OR 3 OR 4 OR 5 OR 6 OR 7 (27057)
8	hospital ADJ1 team* (38)
9	multi-disciplinary team* (258)
10	ward* (MeSH ‘exp Hospitals/) (13153)
11	hospital ADJ1 unit* (372)
12	hospital ADJ1 department* (220)
13	hospital* (MeSH medical personnel/ or health personnel/) (14480)
14	9 OR 10 OR 11 OR 12 OR 13 OR 14 (27498)
15	8 AND 15 (473)

Database: CINAHL <1990 to 2016>	
Search strategy:	
1	reflexiv* (MeSH ‘Reflexivity (Research)) (145)
2	video feedback (373)
3	ethnograph* (MeSH ‘Ethnographic Research/AE/ED/EV/MT/OG/ST’) (54)
4	critical reflection (473)
5	peer assessment (105)
6	simulation training (370)
7	1 OR 2 OR 3 OR 4 OR 5 OR 6 OR 7 (1160)
8	hospital team* (26)
9	multi-disciplinary team* (201)
10	ward* (MeSH ‘MH “Hospital Units”) (4837)
11	hospital ADJ1 unit* (27)
12	hospital ADJ1 department* (27)
13	hospital* (259400)
14	9 OR 10 OR 11 OR 12 OR 13 OR 14 (259731)
15	8 AND 15 (132)

Database: Cochrane Library <1990 to 2016>	
Search strategy:	
1	reflexive (136)
2	video ADJ1 feedback (30)
3	ethnograph* (170)
4	critical reflection (325)
5	peer assess* (6093)
6	simulation (7408)
7	1 OR 2 OR 3 OR 4 OR 5 OR 6 OR 7 (13849)
8	hospital ADJ1 team* (148)
9	multi-disciplinary team* (389)
10	hospital ward* (6482)
11	hospital ADJ1 unit* (319)
12	hospital ADJ1 department* (244)
13	9 OR 10 OR 11 OR 12 OR 13 (7119)
14	8 AND 14 (636)

Database: ISI Web of Science <1990 to 2016>	
Search strategy:	
1	reflexiv* (22412)
2	video*feedback (8723)
3	ethnograph* (44150)
4	critical reflect* (4603)
5	peer assessment* (1758)
6	simulation training (2850)
7	1 OR 2 OR 3 OR 4 OR 5 OR 6 OR 7 (74396)
8	hospital team* (308)
9	multi-disciplinary team* (5859)
10	hospital ward* (3388)
11	hospital unit* (8072)
12	hospital department* (6683)
13	9 OR 10 OR 11 OR 12 OR 13 (23947)
14	8 AND 14 (141)

Database: PubMed	
Search strategy:	
1	reflexiv*
2	video feedback
3	ethnograph*
4	critical reflect*
5	peer assessment* (MeSH terms - exploded)
6	patient simulation* (MeSH terms - exploded)
7	1 OR 2 OR 3 OR 4 OR 5 OR 6 OR 7 (18147)
8	hospital team*
9	multi-disciplinary communication* (MeSH terms - exploded)
10	hospital ADJ1 ward*
11	hospital ADJ1 unit*
12	hospital ADJ1 department*
13	hospital (MeSH terms - exploded)
14	9 OR 10 OR 11 OR 12 OR 13 OR 14 (477734)
15	8 AND 15 (1085)
