# Supplementary material for: Does team reflexivity impact teamwork and communication in interprofessional hospital-based healthcare teams? A systematic review and narrative synthesis
Source: BMJ Qual Saf. 2020 Jan 7;29(8):672–83. doi: 10.1136/bmjqs-2019-009921 (PMC7398296; doi:10.1136/bmjqs-2019-009921)
Supplement: Supplementary data [file bmjqs-2019-009921supp003.pdf]

Details of data extraction

This file summarises the information extracted through the data extraction forms.

General Information:

- Bibliographic details
- Publication type
- Location of research
- Quality assessment score

Overall study information:

- Study aims
- Care setting
- Rationale
- Time period for study
- Inclusion criteria
- Exclusion criteria
- Sample selection
- Sample size
- Appropriateness of sample
- Data collection methods
- Role of researcher
- Data analysis methods
- Researcher bias
- Limitations
- Outcomes
- Themes
- Conclusions
- Generalisability
- Implications
- Future research

Reflexive component of the study:

- Sample size undergoing reflexive component
- Aim of reflexive feedback session
- Reflexive method used
- Form of facilitation
- Main outcomes of reflexive component of method
