# Supplementary material for: Does team reflexivity impact teamwork and communication in interprofessional hospital-based healthcare teams? A systematic review and narrative synthesis
Source: BMJ Qual Saf. 2020 Jan 7;29(8):672–83. doi: 10.1136/bmjqs-2019-009921 (PMC7398296; doi:10.1136/bmjqs-2019-009921)
Supplement: Supplementary data [file bmjqs-2019-009921supp004.pdf]

Appendix D

Extended table of results identifying the key reflexive methods used and the outcomes of collective reflexive discussion.

Author and year	Study setting	Team type	Reflexive method used	Main data collection methods	Main outcomes (Hard and soft outcomes differentiated)	Aim of reflexive feedback session	Facilitation of discussion	Main outcomes of reflexive feedback session
**Allan, Thiagarajan, Beke et al. (2010)**	24-bed dedicated paediatric cardiac intensive care unit (ICU).<br><br>USA	Paediatric cardiac intensive care teams;<br>Nurses (n = 127)<br>Cardiology, cardiac surgery & cardiac critical care fellows (n = 44)<br>pCICU attending physicians (n = 6)<br>Respiratory therapists (n = 2)<br>Nurse practitioners (n = 3)	Simulation based crew resource management (CRM) training	*Quant*: pre- and post-course evaluation questionnaires	**Hard outcomes**<br>Course scored highly on usefulness and realism.<br>After the course participants reported higher confidence and lower anxiety about involvement in future code events. Participants reported increased likelihood of reporting inappropriate management of code events.<br>**Soft outcomes**<br>None reported.	Exploration of efficacy of teamwork and adherence to CRM during the simulated resuscitations.<br>Discussions of medical management.	Video-based debriefing facilitated by nurses and physicians trained in facilitation.	Participants reported feeling more confident in participating in or leading future resuscitation events following exploration of issues raised in debriefing.
**Aveling, Martin, Garcia et al. (2012)**	Lung cancer teams in 30 NHS hospitals.<br><br>UK	Lung cancer teams with a minimum requirement of;<br>A clinical lead (physician)<br>A clinical nurse specialist<br>An MDT coordinator	Reciprocal peer review	*Qual*: ethnographic methods (observations, interviews)	**Hard outcomes**<br>Five key features to optimise reciprocal peer review identified; peers & pairing method, minimising logistic burden, structure of visits, independent facilitation and credibility.<br>**Soft outcomes**<br>Reciprocal peer review generally a positive experience for participants. Implementing	Peer-supported generation of locally-appropriate solutions to issues.	Discussion structured to include direct peer-to-peer discussion, then discussion within teams, then feedback to/from the paired team, steered by an independent facilitator.	Health care practitioners (HCPs) involved could discuss strategies for improvement with peers and identify ways in which improvements could be made.

Appendix D

Extended table of results identifying the key reflexive methods used and the outcomes of collective reflexive discussion.

					improvement plans challenging and requires substantial support.			
**Carroll, Iedema & Kerridge (2008)**	ICU in a tertiary referral and teaching hospital.<br><br>Australia	ICU teams including trainee specialists, specialist intensivists, nurses and allied health professionals (AHPs).	VRE	*Qual*: ethnographic methods (video footage, observations)	**Hard outcomes**<br>Changes to the ward rounds and planning meetings within 2 weeks of the reflexive feedback session. The ward round was split in two to reduce the burden of communication which raised the medical presence on the ground and enhanced interprofessional communication. The daily planning meeting was moved from immediately after the ward round, freeing time for staff to discuss clinical priorities for the morning.<br>A daily worksheet enabling organised review of each patient was finalised and distributed.<br>**Soft outcomes**<br>Watching footage of own practice had dramatic effect on how own practice is experienced.	Visualise the purpose, length and complexity of clinical meetings, and to allow clinicians to articulate the complexities of the clinical communications, and identify solutions to such challenges.	Facilitation was via the primary researcher, who asked questions developed through coding of the original video footage.	Ward round and daily planning meetings were restructured and new documentation system was implemented.
**Falcone, Daugherty, Schweer et al. (2008)**	Paediatric trauma unit in Level 1 paediatric trauma centre.	Paediatric trauma teams including;<br>Paediatric surgeons (n = 11)<br>Emergency medics (n = 7)<br>Surgical residents (n = 72)	Simulation training	*Quant;* pre- and post-training scoring of trauma simulations by independent	**Hard outcomes**<br>Significant improvement in overall performance as determined by the percentage of possible appropriate and timely	Formal debriefing following video review of trauma simulation. Emphasis on team	There is no discussion about the level of facilitation.	Immediate improvement as a result of feedback during debriefing in all groups between first and

Appendix D

Extended table of results identifying the key reflexive methods used and the outcomes of collective reflexive discussion.

	USA	Nurses (n = 60)<br>Critical care fellows (n = 4)<br>Paramedics (n = 2)<br>Respiratory therapists (n = 4)<br>Average team of around 6 members.		reviewers on specific scoring scale.	care measures achieved. Evidence of improvement in airway management, initial trauma assessment, cervical spine precautions and pelvic fracture recognition and management as scored by two blind reviewers. Improvement was shown for teams progressing from their first to second simulated scenario after debrief.<br>**Soft outcomes**<br>Teams in the later groups scored significantly higher than early teams due to improvement in culture over time.	performance and communication.		second scenario scores.
**Fransen, Ven, Schuit et al. (2012)**	Obstetric unit<br>Netherlands	Multi-professional obstetric teams including gynaecologists/obstetricians, secondary care midwives and/or resident nurses.	Simulation training	*Mixed*: video footage, feedback sessions, Clinical Teamwork Scale (CTS).	**Hard outcomes**<br>The composite outcome of obstetric complications didn't differ between study groups. Team training reduced trauma due to shoulder dystocia and increased invasive treatment for severe postpartum haemorrhage.<br>**Soft outcomes**<br>None reported.	Feedback on teamwork and the application of medical technical skills.	There is no discussion about the level of facilitation.	Team training associated with higher Clinical Teamwork Scale score.
**Hor, Iedema & Manias (2014)**	Two general ICUs in a major metropolitan teaching hospital.	ICU staff including senior and junior doctors, senior and junior nurses, medical and nurse managers, ward clerks, receptionists and AHPs.	VRE	*Qual*: ethnographic methods (interviews, video footage, observations)	**Hard outcomes**<br>Two solutions were developed targeting two open spaces where activities were often interrupted unsafely.	To think about how the spaces in their unit impacted on their communication practices with	Facilitated by the researcher, who primed the participants, showed them video clips, and directed	Staff adopted two spatial solutions after devising them in focus groups.

Appendix D
Extended table of results identifying the key reflexive methods used and the outcomes of collective reflexive discussion.

	Australia				Nurses created a new policy restricting interruptions whilst working at the controlled drug cupboard. Doctors moved their X-ray rounds into a new protected space to limit noise and interruptions.<br>**Soft outcomes**<br>Clinicians use space to manage the complexity and safety of their work. The manipulation of space is a case of creating spaces for use through policies, temporary barriers and behaviours.	one another, and identify solutions.	discussion using pre-defined open-ended questions.	
**Iedema, Ball, Daly et al. (2012)**	Emergency departments (ED) of two large teaching hospitals (one metropolitan, one regional).<br>Australia	Paramedics and emergency department medics and nursing clinicians.	VRE	*Qual*: ethnographic methods (focus groups, video footage, observations)	**Hard outcomes**<br>Uptake of new IMIST-AMBO protocol for non-trauma and trauma handovers. This led to a greater volume of information per handover that was more consistently ordered, fewer questions from ED staff, reduction in handover duration, and fewer repetitions by paramedics and ED clinicians.<br>**Soft outcomes**<br>There was an overall increase on agreement in perceived handover improvement post-intervention.	To form and articulate views about what is essential information needing to be communicated, critical process steps to be included in handover, and context characteristics to be maintained.	There is no discussion about the level of facilitation.	What essential information needs to be communicated during ambulance to ED handover, what critical process steps should be included in a new protocol, and what context characteristics should be maintained.
**Iedema & Carroll (2011)**	Acute outpatient spinal clinic in a	Multi-disciplinary care team including doctors, nurses,	VRE	*Qual*: ethnographic	**Hard outcomes**	To enable clinicians to	There is no discussion about	Staff-led redesign of existing ways

Appendix D

Extended table of results identifying the key reflexive methods used and the outcomes of collective reflexive discussion.

	local metropolitan teaching hospital.<br><br>Australia	occupational therapists, physiotherapists, dieticians, social workers and peer support workers.		methods (interviews, video footage, observations)	Systems redesigned following viewing of project footage. Delays or cancellations in surgery targeted by putting in place an alternative pathway involving an agreement to move patients to a neighbouring hospital. Detours in infection control were pre-empted by involving the infection control nurse more closely in scrutinising infection control practices.<br><br>**Soft outcomes**<br><br>Clinicians were able to articulate problems that had thus far not been articulated, and through that, identified ways of tackling problems.	develop a discourse about existing practices and processes, and enabling staff to rethink and redesign existing ways of working.	the level of facilitation.	of working within the unit.
**Iedema, Hor, Wyer et al. (2015)**	ICU and mixed surgical wards in two metropolitan teaching hospitals.<br><br>Australia	107 nurses, 44 doctors, 9 AHPs and 17 administration and cleaning staff.	VRE	*Qual*: ethnographic methods (interviews, video footage, observations)	**Hard outcomes**<br><br>Design of site-specific solutions for future transfer of MRSA-colonised ICU patients through the ward.<br><br>**Soft outcomes**<br><br>Individuals became more aware of theirs and others' care practices.	Allow clinicians to consider infection control practices from different perspective, and articulate solutions to potential issues.	Facilitation of feedback session by researcher using open ended questions and prompts.	Identified previously unrecognized risk in own practice. Formulated safer ways of dealing with infection risks.
**Iedema, Long, Forsyth & Lee (2006)**	Acute outpatient spinal pressure area clinic in a local metropolitan	Medical, nursing and allied health staff working in the outpatients unit.	VRE	*Qual*: ethnographic methods (video footage, observations)	**Hard outcomes**<br><br>A decrease per patient admission from $198,000 to $42,000.	To allow clinicians to identify previously unrecognized	There is no discussion about the level of facilitation.	Redesigning of practices and processes following production and

Appendix D

Extended table of results identifying the key reflexive methods used and the outcomes of collective reflexive discussion.

	teaching hospital.<br><br>Australia				An overall reduction in spending of $600,000 over three years, despite almost double the number of patients treated.<br>**Soft outcomes**<br>Video data allowed the infection control clinician to identify previously unrecognised environmental risk factors. Production of a process map allowed staff to pinpoint the most common sources of team tensions.	environmental risk factors.		discussion of process map.
**Iedema, Merrick, Rajbhandari et al. (2009)**	ICU<br><br>Australia	Multi-disciplinary teams of healthcare practitioners. Make-up of the teams unspecified.	VRE	*Qual*: ethnographic methods (video footage, observations)	**Hard outcomes**<br>New staff to be oriented to the use of electronic resources used during handover to record and organise patient information.<br>**Soft outcomes**<br>Staff in the unit have developed an interest in and an ability to discuss handover in meta-discursive terms; abstracting the discussion away from the here and now and creating new common ground.	To address the strengths and weaknesses of ICU handover practices.	A researcher was present at the meetings to facilitate the discussion, answer questions and point to issues identified through non-participant observation.	Articulation of insights about improving handover practices, at both a specific and systemic level.
**Lehner, Heimberg, Hoffmann et al. (2017)**	Paediatric trauma unit<br><br>Germany	14 physicians including paediatric surgeons, paediatric intensivists, emergency medics and anaesthetists.<br>4 paediatric nurses.	Simulation training	*Mixed*: Video footage, debriefing, pre- and post-course evaluation surveys.	**Hard outcomes**<br>Overall the simulation course received a very positive evaluation. The detailed debriefings were also positively rated. Feedback within	Discussion of key factors relating to CRM learning objectives following simulated scenario.	Facilitated by two-person, interdisciplinary and multi-professional instructor team.	Feedback within the debriefings important and applicable to the clinical routine.

Appendix D

Extended table of results identifying the key reflexive methods used and the outcomes of collective reflexive discussion.

					the debriefing was found to be important and applicable to the clinical routine.<br>**Soft outcomes**<br>Feeling of individual improvement was reported across almost all categories of medical problems. Perceived improvements were also reported in non-technical skills.			
**Patterson, Geis, Falcone et al. (2013)**	Paediatric emergency department<br><br>USA	All personnel who respond to medical or trauma team activations in emergency department resuscitation bay including;<br>Physicians 51%<br>Nurses 32%<br>Paramedics 4%<br>Respiratory therapists 3%<br>Patient care assistant 4%<br>Other 7%	Simulation training	*Mixed*: observation and video recording to score NTS, online survey.	**Hard outcomes**<br>Rate of LST identification improved post-simulation training from 1 in every 7 simulations, to 1 in every 1.2. No visible improvement in scores on the ANTS behavioural scale over time.<br>**Soft outcomes**<br>78% of staff reported the training as valuable, although 77% reported little or no clinical impact.	Self-assessment and group assessment of performance.<br>Identification, evaluation of and solutions to challenges.<br>Identification of LSTs by facilitator and/or team members.	Facilitated debriefing.	Primary outcome measure was the number and types of LSTs identified during the in situ simulations identified during feedback.
**Patterson, Geis, LeMaster et al. (2013)**	Level 1 paediatric trauma centre.<br><br>USA	All healthcare providers in emergency department including;<br>Faculty and staff physicians<br>Nurses<br>Respiratory therapists<br>Paramedics<br>Patient care assistants<br>Medical residents	Simulation training	*Mixed*: patient safety knowledge assessments, SAQ Teamwork and Safety Climate version, a modification of the Behavioural Markers for Neonatal	**Hard outcomes**<br>A sustained improvement in knowledge scores over baseline, with scores immediately post-intervention significantly higher than those at the 10-month retest period. There was a significant improvement in attitudes and culture	Group assessment of team performance as well as identification and suggestion of solutions to any challenges encountered.	Debriefing was facilitated by one of the researchers, and included group assessment of team performance as well as guided review of the simulation video.	Healthcare practitioners develop teamwork and communication skills, and develop the confidence in those skills.

Appendix D

Extended table of results identifying the key reflexive methods used and the outcomes of collective reflexive discussion.

				Resusctiation Scale, filming of simulations and ED resuscitations.	post-intervention. The number of PSEs on the unit reduced from 12 in 5 years to 2 in the 7 years since the beginning of the project.<br>**Soft outcomes**<br>Participants ranked the value of the training highly.			
**Ross, Anderson, Kodate et al. (2013)**	Tertiary hospital trust providing a range of specialist older persons services.<br>UK	HCAs, nurses, physiotherapists and medical staff involved in the provision of elderly care.	Simulation training	*Mixed*: observations of the programme, confidence rating scales and follow-up interviews with staff.	**Hard outcomes**<br>Staff self-confidence scores improved significantly after human-patient simulation and ward-based exercises.<br>**Soft outcomes**<br>Observations showed enjoyment of the course but some apprehension about the simulation environment. Interview data showed perceived learning about teamwork and patient care.	Aim was to focus reflexively on NTS in clinical practice.	Facilitated by clinicians and trained professionals. 45-minute debrief for every 15-minute scenario, structured around descriptive, analysis and application phases.	HCPs involved benefited from increased self-confidence following simulation training, as well as learning about teamwork and patient care.
