# Supplementary material for: Does team reflexivity impact teamwork and communication in interprofessional hospital-based healthcare teams? A systematic review and narrative synthesis
Source: BMJ Qual Saf. 2020 Jan 7;29(8):672–83. doi: 10.1136/bmjqs-2019-009921 (PMC7398296; doi:10.1136/bmjqs-2019-009921)
Supplement: Supplementary data [file bmjqs-2019-009921supp005.pdf]

Appendix E. Quality Assessment Tool (QATSDD) scores for all reviewed papers (scores range from 0 – 3)

Citation Number	Item 1	Item 2	Item 3	Item 4	Item 5	Item 6	Item 7	Item 8	Item 9	Item 10	Item 11	Item 12	Item 13	Item 14	Item 15	Item 16	Score	%
**46**	3	3	3	3	3	3	3	1	0	3	N/A	3	3	N/A	1	3	35/42	83%
**39**	3	3	1	2	3	3	2	2	N/A	N/A	3	3	2	2	0	3	32/42	76%
**25**	3	2	3	0	0	3	3	0	N/A	N/A	3	0	0	0	0	2	19/42	45%
**40**	2	3	1	0	3	3	2	1	3	3	N/A	3	3	N/A	1	3	31/42	74%
**47**	3	3	2	3	3	3	1	2	1	3	N/A	3	3	N/A	0	3	33/42	79%
**36**	3	3	1	0	1	3	3	0	N/A	N/A	3	0	0	0	2	3	22/42	52%
**37**	3	3	1	0	2	3	1	3	0	3	3	2	1	0	3	3	31/48	65%
**41**	3	2	3	0	0	3	2	0	N/A	N/A	1	0	0	0	1	2	17/42	40%
**38**	2	2	0	0	2	2	3	1	N/A	N/A	2	1	0	0	2	3	20/42	48%
**42**	2	2	3	0	0	3	3	0	N/A	N/A	2	1	0	0	1	2	19/42	45%
**43**	3	2	0	0	0	3	3	0	N/A	N/A	3	1	0	0	2	2	19/42	45%
**44**	2	3	0	0	1	2	0	0	0	3	N/A	3	1	N/A	0	3	18/42	43%
**45**	3	3	3	0	2	3	3	2	2	3	2	3	2	0	0	3	34/48	71%
**48**	1	3	2	0	2	3	3	1	3	3	N/A	3	1	N/A	2	3	30/42	71%
**49**	3	3	1	0	2	2	2	1	2	3	3	3	1	1	0	2	29/48	60%

Item 1: Explicit theoretical framework
Item 2: Statement of aims/objectives in main report
Item 3: Clear description of research setting
Item 4: Evidence of sample size considered in terms of analysis
Item 5: Representative sample of target group of a reasonable size
Item 6: Description of procedure for data collection
Item 7: Rationale for choice of data collection tool(s)
Item 8: Detailed recruitment data
Item 9: Statistical assessment of reliability and validity of measurement tool(s) (Quantitative studies only)
Item 10: Fit between research question and method of data collection (Quantitative studies only)
Item 11: Fit between research question and format and content of data collection tool e.g. interview schedule (Qualitative studies only)
Item 12: Fit between research question and method of analysis (Quantitative studies only)
Item 13: Good justification for analytic method selected
Item 14: Assessment of reliability of analytic process (Qualitative studies only)
Item 15: Evidence of user involvement in design
Item 16: Strengths and limitations critically discussed
